# High-Resolution Postcontrast Time-of-Flight MR Angiography of Intracranial Perforators at 7.0 Tesla

**DOI:** 10.1371/journal.pone.0121051

**Published:** 2015-03-16

**Authors:** Anita A. Harteveld, Laurens J. L. De Cocker, Nikki Dieleman, Anja G. van der Kolk, Jaco J. M. Zwanenburg, Pierre A. Robe, Peter R. Luijten, Jeroen Hendrikse

**Affiliations:** 1 Department of Radiology, University Medical Center Utrecht, Utrecht, The Netherlands; 2 Image Sciences Institute, University Medical Center Utrecht, Utrecht, The Netherlands; 3 Department of Neurosurgery, University Medical Center Utrecht, Utrecht, The Netherlands; Charité University Medicine Berlin, GERMANY

## Abstract

**Background and Purpose:**

Different studies already demonstrated the benefits of 7T for precontrast TOF-MRA in the visualization of intracranial small vessels. The aim of this study was to assess the performance of high-resolution 7T TOF-MRA after the administration of a gadolinium-based contrast agent in visualizing intracranial perforating arteries.

**Materials and Methods:**

Ten consecutive patients (7 male; mean age, 50.4 ± 9.9 years) who received TOF-MRA at 7T after contrast administration were retrospectively included in this study. Intracranial perforating arteries, branching from the parent arteries of the circle of Willis, were identified on all TOF-MRA images. Provided a TOF-MRA before contrast administration was present, a direct comparison between pre- and postcontrast TOF-MRA was made.

**Results:**

It was possible to visualize intracranial perforating arteries branching off from the entire circle of Willis, and their proximal branches. The posterior cerebral artery (P1 and proximal segment of P2) appeared to have the largest number of visible perforating branches (mean of 5.1 in each patient, with a range of 2–7). The basilar artery and middle cerebral artery (M1 and proximal segment M2) followed with a mean number of 5.0 and 3.5 visible perforating branches (range of 1–9 and 1–8, respectively). Venous contamination in the postcontrast scans sometimes made it difficult to discern the arterial or venous nature of a vessel.

**Conclusion:**

High-resolution postcontrast TOF-MRA at 7T was able to visualize multiple intracranial perforators branching off from various parts of the circle of Willis and proximal intracranial arteries. Although confirmation in a larger study is needed, the administration of a contrast agent for high-resolution TOF-MRA at 7T seems to enable a better visualization of the distal segment of certain intracranial perforators.

## Introduction

Intracranial perforating arteries and small branches of the circle of Willis provide blood supply to the brain stem and deep grey and white matter structures of the brain [1, 2]. Acute occlusion of these perforating arteries may cause infarctions with profound clinical symptoms, while chronic small vessel disease of the perforating arteries may cause chronic white matter lesions in the pons and the cerebrum [3–7]. Due to the size of these small perforating arteries (<1.1 mm in diameter [1]), a high spatial resolution is required for clear visualization of these vessels and their pathology. With the spatial resolution of magnetic resonance angiography (MRA) at standard field strengths (1.5 or 3.0 tesla (T)), usually only the larger intracranial arteries can be visualized [8]. Therefore, in current clinical practice, intra-arterial digital subtraction angiography (iaDSA) is used to visualize smaller arteries, including the perforating arteries. Disadvantages of iaDSA are its use of ionizing radiation, its invasiveness [8], and its inability to visualize the brain parenchyma.

Visualizing perforating arteries with MRI allows for a combined evaluation of vascular lesions and the consequences of these lesions on the brain parenchymal level (such as infarcts and white matter lesions). Ultrahigh-field MRI, like 7T, has the advantage of an increased SNR compared with 1.5T and 3T MRI, enabling a higher spatial resolution within clinically feasible scan times (<10 minutes). Also, the *T*_*1*_ relaxation time of tissues increases with higher field strengths, yielding a better contrast between suppressed background and flowing blood in time-of-flight MRA (TOF-MRA). Due to the longer *T*_*1*_, spins in static tissue show decreased relaxation in between the radiofrequency pulses, resulting in lower signal of static tissue [9]. Different studies already demonstrated the benefits of 7T for TOF-MRA in the visualization of intracranial small vessels [8, 10–21].

In previous reports, 7T MRI has been shown to be capable of visualizing the lenticulostriate arteries [13, 15, 17–21], perforating arteries originating from the posterior communicating artery (PCoA) [14], and basilar artery (BA) perforators [16]. These previous studies, in which each individual study focused on the visualization of one type of perforators only, were performed using TOF-MRA techniques without contrast administration. The lack of contrast administration in TOF-MRA may predispose to signal loss due to saturation effects, particularly in vessels with slow blood flow (like small arteries) [22]. Furthermore, more complex flow patterns might be present in patients with vascular lesions leading to changed signal properties [11]. Contrast administration shortens the *T*_*1*_ relaxation time of blood and thereby might improve the contrast-to-noise ratio between the blood and the surrounding brain parenchyma, giving a clearer depiction of slow-moving blood in distal vessels [22, 23]. However, the contrast agent also shortens the *T*_2_^*^ relaxation time, which could reduce visibility of the blood vessels. Although previous studies have already demonstrated better visualization of the distal segments of large intracranial arteries after the administration of a contrast agent at 1.5T and 3T TOF-MRA [22–24] as well as 7T MPRAGE MRA [25], the value of contrast administration has never been evaluated at 7T TOF-MRA for the smaller intracranial perforating arteries.

The aim of the current study was to assess the visualization of intracranial perforators with high-resolution 7T TOF-MRA after the administration of a gadolinium-based contrast agent.

## Materials and Methods

### Study population

This retrospective study was approved by the local medical ethics committee of University Medical Center Utrecht. All patients clinically imaged between October 2012 and April 2013 at our institution, who underwent a postcontrast 7T TOF-MRA were included. All included subjects gave written informed consent.

### MR imaging protocol

Imaging was performed on a whole-body human 7T MR system (Philips Healthcare, Cleveland, OH, USA) equipped with a 32-channel receive head coil and volume transmit/receive coil for transmission (Nova Medical, Wilmington, MA, USA). A patient-specific clinical imaging protocol was obtained in all patients. Before acquisition of the TOF-MRA sequence, 0.1 mmol/kg of a gadolinium-containing contrast agent (Gadobutrol, Gadovist 1.0mmol/mL, Bayer Schering Pharma, Newbury, UK) was administered intravenously to the patient. The following imaging parameters were used for the TOF-MRA sequence: FOV 200 x 190 x 50 mm^3^ in transverse orientation, acquired voxel size 0.25 x 0.3 x 0.4 mm^3^, TR 15.3 ms, TE 3.4 ms, flip angle 25 degrees, number of slices 250, acquisition time approximately 9 minutes. A high-resolution Fluid-Attenuated Inversion Recovery (FLAIR) sequence, for anatomical verification, was used with the following imaging parameters: FOV 250x250x190 mm^3^, acquired voxel size 0.8x0.8x0.8 mm^3^, TR/TE/TI 8000/300/2250 ms, flip angle 100 degrees, number of slices 475, acquisition time approximately 13 minutes.

### Processing & image analysis

For analysis of the acquired 3D TOF-MRA data, MPR (multi planar reconstruction; thickness 0.4mm; no overlap) and MIP (maximum intensity projection; thickness 10mm; 8mm overlap) slabs were made in coronal, sagittal and transverse orientations on an offline workstation (Philips). A descriptive, qualitative analysis of the location and number of perforating arteries was performed. Perforating arteries originating directly from the following feeding vessels were evaluated: the anterior, middle and posterior cerebral arteries (A1, M1, P1 and proximal part of A2, M2 and P2); the anterior communicating artery (ACoA); the anterior choroidal artery (AChA); the posterior communicating artery (PCoA); the entire basilar artery (BA); and the distal vertebral artery (VA). Most perforating arteries arise from these vessel segments [1]. The lenticulostriate arteries were divided into a medial group, originating from the anterior cerebral artery, and a lateral group, originating from the middle cerebral artery [26]. Perforators were grouped according to the parent artery they originated from (including left or right) and the direction or target of the perforator. Because of the relatively limited FOV, in some scans the more caudal part of the circle of Willis (distal BA and/or VA) was not included (distal BA and VA: 3 scans; VA: 5 scans). The FLAIR images were used for anatomical assessment of direction or target brain region of the perforators. MPR slabs (thickness 0.8mm, no overlap) were made in coronal, sagittal and transverse orientation. The perforating arteries were scored by 2 observers (AH and LDC).

### Comparison with precontrast MRA

In two patients also a TOF-MRA without contrast administration was available, acquired with the same imaging parameters and image orientation. This allowed for an additional comparison between the perforating arteries as visualized on the post- versus precontrast TOF-MRA in the same patient.

## Results

### Study population

In total 12 consecutive patients were eligible for this study, however, two patients were excluded due to motion artifacts, resulting in a total of 10 patients (7 male; mean age 50.4 ± 9.9 years). The patient population consisted of patients with suspected cerebral vasculitis (n = 4), suspected reversible cerebral vasoconstriction syndrome (n = 1), pre-operative assessment of the anatomical relation between brain tumor and arterial perforators (n = 2), subarachnoid hemorrhage (n = 2) and suspected (but undefined) cerebrovascular disease (n = 1).

### Postcontrast TOF-MRA

**Fig 1:**
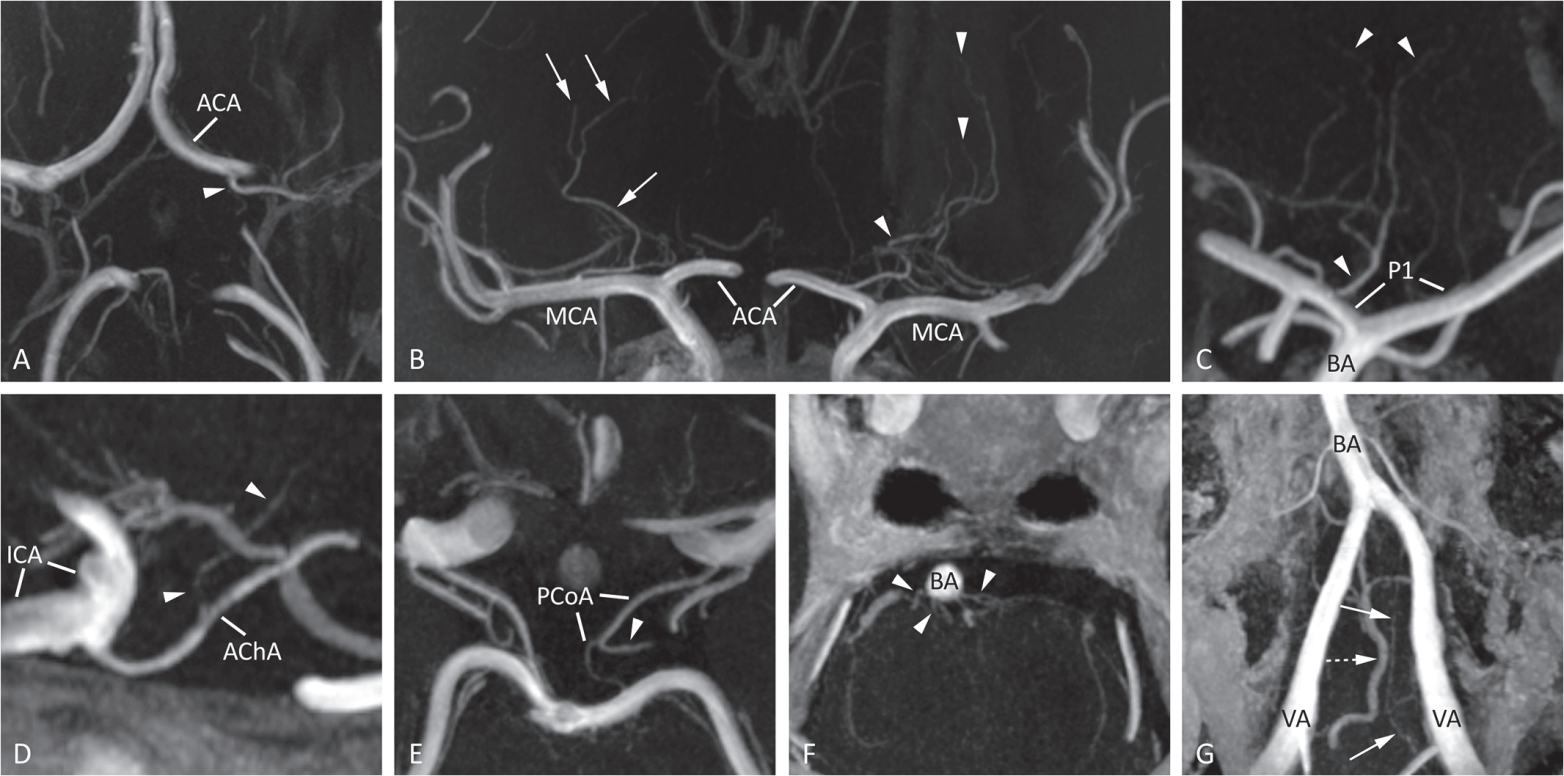
Typical images of intracranial perforators from different patients, obtained by postcontrast TOF-MRA at 7T. (A) A medial lenticulostriate artery (arrowhead), arising from the A1 segment of the ACA (transverse slab MIP, thickness 10mm), (B) lateral lenticulostriate arteries arising from the right MCA (arrows) and medial lenticulostriate arteries arising from the left ACA (arrowheads; coronal slab MIP, thickness 10 mm), (C) artery of Percheron (arrowheads), arising from the P1 segment of the PCA (coronal slab MIP, thickness 10mm), (D) perforating branch (arrowheads) arising from the right AChA (sagittal slab MIP, thickness 10mm), (E) thalamoperforating artery (arrowhead), arising from the left PCoA (transverse slab MIP, thickness 6mm), (F) pontine arteries (arrowheads) arising from the BA (transverse slab MIP, thickness 4mm), and (G) the intracranial feeders of the anterior spinal artery (arrows) with an adjacent vein (dashed arrow, transverse slab MIP angulated anterior-posterior in line with the BA, thickness 10mm). ACA = anterior cerebral artery; AChA = anterior choroidal artery; BA = basilar artery; ICA = intracranial carotid artery; MCA = middle cerebral artery; MIP = maximum intensity projection; PCA = posterior cerebral artery; PCoA = posterior communicating artery; P1 = first segment of the PCA; VA = vertebral artery.

Typical images of intracranial perforators from different patients, obtained by postcontrast TOF-MRA at 7T, are shown in Fig. 1. The high spatial resolution in all three anatomical planes (0.25 x 0.3 x 0.4 mm^3^) enabled reconstructions without loss of spatial resolution. An overview of the identified perforating arteries branching off from the circle of Willis and proximal intracranial arteries on the postcontrast TOF-MRA scans is given in Table 1. A large variety in number and location of the perforating branches between patients could be seen. An overview of the number (mean and range) of perforating branches per parent artery is shown in Table 2. The PCA (P1 and proximal segment of P2) appeared to have the largest number of visible perforating branches (mean, 5.1; range, 2–7). The BA and MCA (M1 and proximal segment M2) followed with a mean number of 5.0 and 3.5 visible perforating branches (range, 1–9 and 1–8, respectively).

**Table 1:** Overview of the number of perforating arteries branching off from the circle of Willis and proximal intracranial arteries identified in the individual postcontrast TOF-MRA scans (1–10).

		1		2		3		4		5		6		7		8		9		10	
Parent artery	Target / direction (name)	# L	# R	# L	# R	# L	# R	# L	# R	# L	# R	# L	# R	# L	# R	# L	# R	# L	# R	# L	# R
ACoA	unknown			1								1				1				1	
ACA	medial lenticulostriate arteries	1 (A1/A2)		1 (A1)	1 (A1)	1 (A2)		1 (A1)	1 (A2)	1 (A2)	1 (A1/A2)	2 (A1)	1 (A1)		1 (A1/A2)	2 (A1)	2 (A2)	1 (A1)	1 (A1)	2 (A1 + prox A2)	1 (A1)
	unknown										1 (A2)	2 (A1)	1 (A1)								
MCA	lateral lenticulostriate arteries		1 (M1)	1 (M2)	1 (M1)	M1 L absent	1 (M1)	3 (M1)	2 (M1)	2 (M1)	1 (M1)	2 (M1)	2 (M1)	3 (M1)	2 (M1)	1 (M1)	2 (M1)	3 (M1)	2 (M1)	2 (M1)	1 (M1)
	unknown							1 (M1)	2 (M1)												
AChA	internal capsule								1												
	unknown	1	1	1	3	2	1	1	1	1		2	2			2	2		1		
PCoA	various (hypothalamus / optic tract / thalamus)		1				PCoA R absent	PCoA L absent		1						1	1	PCoA L absent	PCoA R absent	1	1
	unknown			1	1	1						1	2	1	1						
PCA	interpeduncular cistern / peduncle	1 (P1)		1 (P1)	1 (P1)	2 (P1)	3 (P1)	1 (P1)		2 (P1)						2 (P1)	1 (P1)		1 (P1)	2 (P1)	
	midbrain (circumflex arteries)	2 (P2 +P1)	1 (P2)	2 (P1+P2)	1 (P1)	1 (P1)	1 (P1)	1 (P1)	2 (P1)	1 (P1)	2 (P1)	2 (P1)	2 (P1 + P2)		1 (P1)	1 (P1)	1 (P1)	1 (P1)	2 (P1 + P1/P2)	1 (P1)	2 (P1)
	both thalami (Percheron)				1 (P1)																1 (P1)
	thalamus							1 (P1)	1 (P1 + BA/P1)				1 (P1)								
	lentiform nucleus																			1 (P1)	
	unknown														1 (P1)						
BA	pontine	8		(*)		(*)		4 (*)		4		2		1		7		8 (7x BA, 1x SCA L)		(*)	
	midbrain																	1 (SCA R)			
VA	feeder of anterior spinal artery	1	1	(**)		(**)		(**)		(**)		(**)		(***)			2	(**)		(**)	

(*) only distal segment BA in FOV;

(**) VA not in FOV;

(***) only distal segment VA L in FOV.

A2, M2 and P2:only proximal segment taken into account. L = left; R = right. A1, M1, P1 = first segment of the anterior, middle and posterior cerebral artery; A2, M2, P2 = second segment of the anterior, middle, and posterior cerebral artery; ACoA = anterior communicating artery; ACA = anterior cerebral artery; AChA = anterior choroidal artery; BA = basilar artery; MCA = middle cerebral artery; PCA = posterior cerebral artery; PCoA = posterior communicating artery; SCA = superior cerebellar artery; VA = vertebral artery.

**Table 2:** Mean number and range of perforating branches per parent artery identified in the 7T postcontrast TOF-MRA images.

Parent artery	Mean (number of perforators/ number of patients	Range
Anterior communicating artery	0.4 (4/10)	0–1
Anterior cerebral artery	2.5 (25/10)	1–4
Middle cerebral artery	3.5 (35/10)	1–8
Anterior choroidal artery	2.2 (22/10)	0–4
Posterior communicating artery^a^	1.6 (14/9)	1–3
Posterior cerebral artery	5.1 (51/10)	2–7
Basilar artery^b^	5.0 (35/7)	1–9
Vertebral artery^b^	2.0 (4/2)	2

^a^In one patient the posterior communicating artery was absent bilaterally.

^b^Only scans containing this segment within the FOV were taken into account.

Perforators are grouped based on the parent artery they originate from (including left of right), and their target or direction. Some identified perforating arteries were classified to have 'unknown' target or direction because of incomplete visualization and/or lack of visible anatomical landmarks on the TOF-MRA scan.

### Comparison with precontrast TOF-MRA

**Fig 2:**
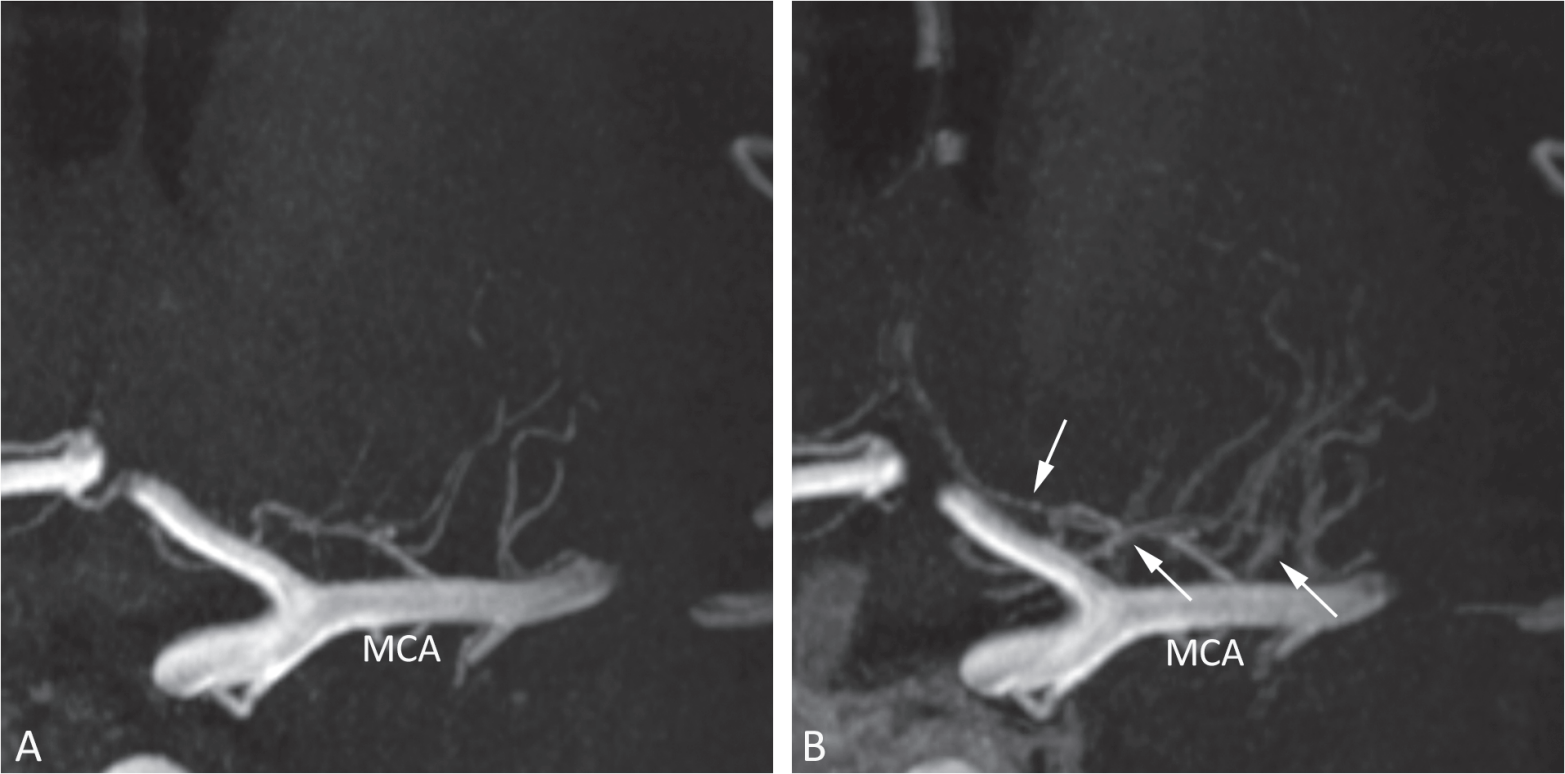
7T TOF-MRA, coronal slab MIP images (thickness 10mm) of the perforating arteries arising from the left MCA before (A) and after (B) contrast administration. In B, the effect of venous enhancement (arrows) can be appreciated, making differentiation between small arteries and veins more difficult. MCA = middle cerebral artery; MIP = maximum intensity projection.

**Fig 3:**
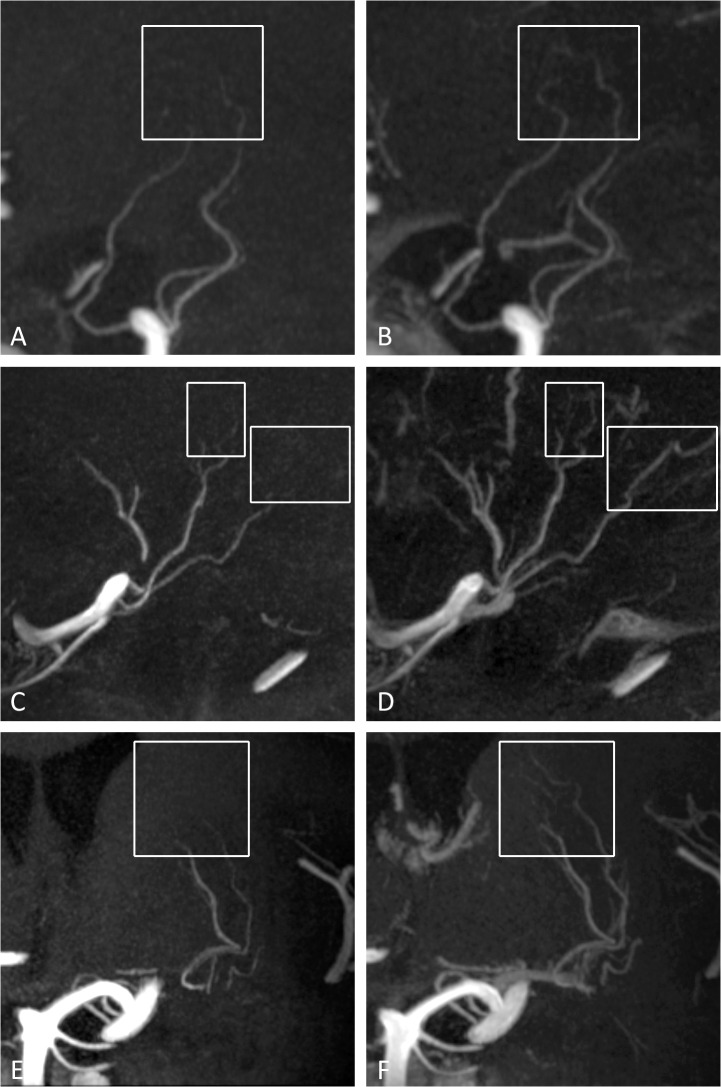
7T TOF-MRA MIP images of different intracranial perforating arteries. **On the left (A,C,E) the precontrast images with the corresponding postcontrast images of the same patient on the right (B,D,F)**. In B, D and F, longer arterial trajectories can be seen after contrast administration as compared to the corresponding unenhanced image in A, C and E (white boxes). (A and B) Sagittal slab MIP, thickness 10mm; (B and C) sagittal slab MIP, thickness 10mm; (E and F) coronal slab MIP, thickness 10mm. MIP = maximum intensity projection.

No differences were found in the observed number of perforating arteries branching off from the parent arteries of the circle of Willis and proximal intracranial arteries before and after administration of the contrast agent. However, interpretation of the postcontrast images for identifying the perforating arteries was found to be more difficult compared to the precontrast images, especially for the perforators originating from the MCA and BA, because of adjacent venous enhancement in the images after contrast administration. An example of this venous enhancement can be seen in Fig. 2. On the other hand, the most distal segments of some perforating arteries could be followed over a longer trajectory after the administration of the contrast agent (Fig. 3).

## Discussion

The current study shows that high-resolution 7T TOF-MRA combined with a gadolinium-based contrast agent clearly visualizes intracranial perforating arteries branching off from the circle of Willis and proximal intracranial arteries. Unlike foregoing studies [13–21], we did not restrict our study to a particular set of perforators, but aimed to investigate the perforating branches of the entire circle of Willis and the proximal intracranial arteries.

When performing a TOF-MRA scan after contrast administration, veins will enhance in addition to arteries. For some clinical applications, e.g. preoperative assessment of tumor resectability, knowledge of venous anatomy in addition to arterial anatomy may be advantageous. A previous study [27] proposed a susceptibility-weighted angiography (SWAN) sequence that allowed visualization of both cerebral veins and arteries in one sequence without application of a contrast agent. Nevertheless, SWAN was found to be clearly inferior to TOF-MRA in the depiction of small arteries. Postcontrast TOF-MRA might have the potential to image both the very small arteries as well as the venous vasculature.

Our study revealed several disadvantages of using a contrast agent for TOF imaging. For some perforators the origin from the parent artery was obscured by the close proximity of veins in the postcontrast images. This sometimes made it difficult to discern the arterial or venous nature of a vessel, while the absence of venous enhancement facilitated the detection of intracranial perforators in the scans before contrast administration. Combining both techniques (performing both the pre- and postcontrast scan) may solve this problem [28], given there is sufficient time for this in the scan protocol and the patient is able to lie still.

Although some perforators could be followed over a longer trajectory with postcontrast TOF-MRA compared to precontrast, no differences were found in the number of visualized perforators within the same patient. Our results regarding the visualization of small intracranial arteries over a longer trajectory after contrast administration corresponds to previous study results. [23–25]. Özsarlak et al, using 1.5T TOF-MRA with low-dose contrast administration, showed improved visualization and assessment of distal branches of the intracranial arteries, while precontrast MR angiograms were found to be superior to low-dose postcontrast MR angiograms for visualization of the circle of Willis and proximal branches. Yano et al. also showed the usefulness of contrast administration for visualizing the distal arterial branches and venous structures at 1.5T. Umutlu et al. evaluated a pre- versus postcontrast MPRAGE sequence at 7T, and found that contrast administration particularly increased the conspicuity of peripheral vessels as well as the complete posterior circulation. This increased conspicuity of the posterior circulation was not seen in our study, possibly because of our relatively limited FOV which could not always include the entire posterior circulation.

The number of visualized perforators was not increased after contrast administration. However, since these results are based on 2 patients in the current study, they will need to be confirmed in a larger study. The TOF-MRA sequence before and after contrast administration was performed with the same imaging parameter settings. With optimization (e.g. shorter TR, larger flip angles) of the postcontrast TOF-MRA for the new T_1_ relaxation of blood, even better contrast may be obtained with shorter acquisition times, or increased spatial resolution. This may allow for visualization of intracranial perforators which are now below the detection limit of the current sequence, since our sequence only has a spatial resolution of 0.25 x 0.3 x 0.4 mm, whereas diameters of intracranial perforators have been reported to range from 70–1150 μm (postmortem data) [1].

Clinical opportunities for postcontrast TOF-MRA could include the pre-treatment evaluation of perforators branching from aneurysms, as well as the effects of (flow-diverting) stents which may or may not cause blockage of small perforating arteries in the proximity of aneurysms. In tumor patients, the small vessels that run in close proximity to brain tumors may be identified [29, 30]. And in patients with small vessel disease (stenosis or occlusion of small arteries), assessment of these small perforating arteries might show the extent and severity of lipohyalinosis, provided that the size of these arteries or arterioles would be above the detection range. Postcontrast TOF-MRA seems to have the advantage of better visualization of the distal segments of the intracranial (perforating) arteries, possibly beneficial for e.g. tumor resection planning or identification of a distal thrombus. Therefore, in each patient, a compromise has to be made whether better visualization of the distal intracranial (perforating) arteries or the proximal intracranial arteries is more important.

This study has limitations. First, scan time of this sequence is relatively long due to its high spatial resolution, necessitating a relatively small coverage and increasing the likelihood of movement artifacts. The small FOV sometimes made it difficult to include both the entire circle of Willis and the more distal branches. Second, not all perforators of the circle of Willis and proximal intracranial arteries could be visualized, since the number of visualized perforating arteries is still much lower than that found in postmortem studies[1]; for instance, for the MCA a mean of 8.6 with a range of 3–15 perforating branches were found in the postmortem study[1], compared to a mean of 3.5 with a range of 1–8 in our study. Further, this study describes the results from a small number of (heterogeneous) subjects, in which only 2 subjects had precontrast TOF-MRA available for comparison; as already mentioned before, a prospective study with a large sample size is needed to strengthen the main findings of this study.

Part of the variability in the visibility of the perforating arteries may reflect variability in the mean blood flow velocities in these small arteries. Potential improvements for further use of this method in larger studies would be to divide the 3D FOV in multiple (slightly overlapping) 3D thin slabs [31]. This will improve the visibility of relatively slow flowing blood. Besides, more advanced excitation pulses may be used to reduce the SAR and improve the background suppression [32]. Another potential improvement would be to increase the spatial resolution to image intracranial perforators which are now below the detection limit of the used sequence. However, this comes at the cost of decreased signal-to-noise ratios and an increase in scan time, while the scan time of the currently used sequence is relatively long already. Future developments may lead to receive coils with extremely high numbers of receive coils [33], which provides more flexibility for faster imaging or for increasing the FOV within the same scan time. Faster imaging may facilitate the direct comparison between pre- and postcontrast MRI within the same scan session, while an increased FOV could, in addition to the perforating arteries in the current studies, allow for an assessment of peripheral cortical arteries as well as small arteries within the posterior circulation. In addition, to provide better insight in the number of intracranial perforators currently missed with TOF-MRA, a direct comparison could be made with iaDSA in the same patient.

In conclusion, high-resolution postcontrast TOF-MRA at 7T is able to visualize multiple small intracranial perforators with high image contrast in patients. Although confirmation in a larger study is needed, the administration of a contrast agent for high-resolution TOF-MRA at 7T seems to enable a better visualization of the distal segment of certain intracranial perforators.
